# Decreased mean platelet volume and increased platelet to lymphocyte ratio in patients with pterygium

**DOI:** 10.5935/0004-2749.20200069

**Published:** 2020

**Authors:** Raşit Kılıç, Ali Kurt

**Affiliations:** 1 Department of Ophthalmology, Faculty of Medicine, Ahi Evran University, Kırşehir, Turkey

## INTRODUCTION

Pterygium is a common disorder of the ocular surface characterized by collagen degeneration and overgrowth of fibrovascular tissue from the conjunctivae onto the cornea^(1)^. The Neutrophil to lymphocyte ratio (NLR), monocyte to lymphocyte ratio (MLR), platelet to lymphocyte ratio (PLR) and mean platelet volume (MPV) have all been explored as potential biomarkers for inflammation associated with ophthalmic diseases.

In this study, we evaluated MPV, NLR, MLR and PLR and identified specific associations with the pathogenesis of pterygium.

## METHODS

Approval was provided by the Ahi Evran University Ethics Committee (date: 24.01.2017 - number: 201702/15). The study was conducted according to the Declaration of Helsinki. Seventy-one patients diagnosed with pterygium and 46 ageand sex-matched healthy control subjects who presented to our clinic were enrolled in this study.All participants provided written informed consent. Patients with recurrent pterygium, history of any other ocular disorder or surgery including corneal trauma, corneal scarring, glaucoma, uveitis or severe dry eye, and those diagnosed with any systemic disorder, systemic inflammation or clinical condition, pregnancy, ato py, or currently using any topical or systemic medication or anti-inflammatory or antioxidant therapies were excluded from the study. The pterygium patients were classified into three groups, including atrophic, fleshy or intermediate according to Tan’s classification^(2)^. For bilaterally affected cases, more advanced eye was included in the classification.

Neutrophil, lymphocyte, monocyte and platelet counts, and MPV were measured within 2 hours after collection of blood samples with the use of Sysmex XN-1000 analyzer. The statistical methods evaluating the data were the chi-square, independent t test and Spearman’s Rho correlation.The overall accuracy was determined by the area under the curve, sensitivity and specificity using receiver-operating characteristic (ROC) analysis. In all tests, p<0.05 was considered to be statistically significant.

## RESULTS

The mean age of the pterygium patients was 43.4 ± 10.2 years; the mean age of the controls was 43.2 ± 9.0 years (Table 1). We found significantly lower MPVs and higher PLRs among pterygium patients compared to values obtained from age-matched healthy controls (p=0.000 and p=0.032, respectively). Groups included 20 eyes classified as atrophic, 29 eyes classified as intermediate and 22 eyes classified with fleshy pterygium. A significant inverse correlation was found between pterygium classification and MPV (p=0.030 and r=-0.259), although no significant correlations with NLR, MLR or PLR were identified (p>0.05). The results of ROC analysis are shown in table 2.

**Table 1:** Comparison of demographic features and blood parameters between pterygium patients and healthy controls

	Pterygiumpatients (n=71)	Healthy controls (n=46)	p-value
Age	43.4 ± 10.2	43.2 ± 9.0	0.945
Gender (female/male)	35/36	23/23	0.941
MPV (fL)	9.39 ± 1.33	10.45 ± 1.0	0.000^*^
PLR	129.5 ± 39.8	113.3 ± 38.9	0.032^*^
NLR	1.90 ± 0.56	1.73 ± 0.67	0.156
MLR	0.24 ± 0.07	0.23 ± 0.06	0.410

* Statisticallysignificant (p<0.05).

MPV= meanplatelet volume; PLR= platelet to lymphocyte ratio; NLR= neutrophiltolymphocyteratio; MLR= monocytetolymphocyteratio.

**Table 2:** Receiver-operating characteristic (ROC) curve analysis

Parameters	AUC ± SE	Cutoff	Sensitivity	Specificity	p-value
MPV	0.746 ± 0.045	9.85	67	67	0.000^*^
PLR	0.629 ± 0.053	114.5	62	61	0.018^*^
NLR	0.589 ± 0.056	1.76	59	54	0.105
MLR	0.547 ± 0.054	0.23	59	52	0.396

* Statistically significant (p<0.05).

AUC= area under the curve; SE= standart error; MPV= mean platele tvolume; PLR= platelet to lymphocyt eratio; NLR= neutrophil to lymphocyte ratio; MLR= monocyte to lymphocyte ratio.

## DISCUSSION

To the best of our knowledge, this is the first study designed to assess MPV, NLR, MLR and PLR in patients diagnosed with pterygium. Our results demonstrate that the MPV was significantly lower and PLR was significantly higher in pterygium patients than in control subjects. We also determined an inverse correlation between MPV and the pterygium classification groups. ROC analysis indicated that the sensitivity and specificity of MPV and PLR were greater than 60%.

With respect to mechanism, ultraviolet light is well-known to promote local inflammation and corneal damage^(1)^. While the factors that promote the development of pterygium are not completely understood, many proinflammatory cytokines and angiogenic growth factors triggered by ultraviolet light may contribute to this disease^(1)^. Furthermore, Lee et al.^(3)^ reported that ischemic signals from the ocular surface, including pro in flammatory cytokines, diffused into systemic circulation from the pterygium; these systemic cytokines might then have an impact on progenitor cells in the bone marrow microenvironment. Mean platelet volume reflects platelet activation and function and can be directly modulated by inflammation^(4)^; elevated levels of proinflammatory cytokines in system circulation can suppress the size of platelets via their direct impact on megakaryocyte differentiation^(4,5)^. Comparatively large platelets typically release proinflammatory and thrombotic agents more actively than small platelets and there is an overall increased demand for platelets in the setting of acute inflammation^(4,5)^. The results from the present study permit us to hypothesize that increasing levels of proinflammatory cytokines in pterygium may ultimately have an impact on the bone marrow and may promote the production of comparatively smaller platelets. Moreover, we observed that MPV decreased in assocation with an increase in pterygium grade. Likewise, PLR was found to be higher and MPV was found to be lower in pterygium than in other known ocular disorders.

In conclusion, our findings that relate MPV and PLR to the diagnosis and classification of pterygium may provide additional information about inflammatory processes at the cornea.
